# P41 - Preexposure prophylaxis of infants’ food allergy

**DOI:** 10.1186/2045-7022-4-S1-P96

**Published:** 2014-02-28

**Authors:** Svetlana Denisova, Vera Revyakina, Tatiana Sentsova, Marina Belitskaya, Elena Pavlovskaya, Ilia Vorozhko

**Affiliations:** 1 N.I. Pirogov Russian National Research Medical University, Moscow, Russia; 2 Research Institute of Nutrition, Russian Academy of Medical Sciences, Moscow, Russia; 3 G.N. Speransky Municipal Children’s Clinical Hospital No 9, Moscow, Russia

## Background

The problem of food allergy remains actual till now, and demands further development of therapeutical and prevention programs, including diet therapy for lactating mothers. The aim of the work is clinical and immunological evaluating of diet therapy of lactating mothers whose children with atopic dermatitis (AD) were on the exclusive breastfeeding.

## Methods

We observed 100 “mother and child” pairs, which were divided into two groups. All children had AD, associated to cow milk protein (CMP) allergy. Lactating mothers from both groups were treated by diet therapy with the replacement of the cow milk to the New Zealand goat milk (1^st^ group: 43 “mother and child” pairs) or non-dairy diet (2^nd^ group: 57 “mother and child” pairs). The effect of diet therapy was assessed by dynamics of clinical and immunological AD symptoms in infants after 1-3 months of treatment. Immunological effect of the therapy was assessed by the dynamics of levels of allergen-specific IgE and IgG to CMP, casein, β-lactoglobulin and goat milk protein which were measured by uncompetitive immunoenzyme assay on special test-systems from Allergopharma (Germany). The level of IFN-γ, IL-2, IL-4, IL-5, IL-12, IL-13 was measured by immunoenzyme ELISA method.

## Results

During the treatment, that included diet therapy of lactating mothers and anti-allergic therapy of infants, patients of both groups showed remission with saved breastfeeding. Immunological evaluating of therapeutic intervention’s effectiveness revealed the positive dynamics of levels of total IgE, allergen-specific IgE and IgG antibodies to CMP and its fractions, as well as to soy and goat milk protein in the blood of all children. Moreover, complex therapy conduced decreasing of proinflammatory cytokines blood level.

## Conclusion

The obtained results confirmed reasonability of diet treatment of lactating mothers, whose children have AD, associated with CMP allergy.

